# Midwives’ perspectives on person-centred maternity care in public hospitals in South-east Nigeria: A mixed-method study

**DOI:** 10.1371/journal.pone.0261147

**Published:** 2021-12-10

**Authors:** Daniel Chukwuemeka Ogbuabor, Ijeoma Lewechi Okoronkwo

**Affiliations:** 1 Department of Health Administration and Management, University of Nigeria Enugu Campus, Enugu, Enugu State, Nigeria; 2 Department of Health Systems and Policy, Sustainable Impact Resource Agency, Enugu, Enugu State, Nigeria; Michigan State University College of Human Medicine, UNITED STATES

## Abstract

**Background:**

Person-centred maternity care (PCMC) is acknowledged as essential for achieving improved quality of care during labour and childbirth. Yet, evidence of healthcare providers’ perspectives of person-centred maternity care is scarce in Nigeria. This study, therefore, examined the perceptions of midwives on person-centred maternity care (PCMC) in Enugu State, South-east Nigeria.

**Materials and methods:**

This study was conducted in seven public hospitals in Enugu metropolis, Enugu State, South-east Nigeria. A mixed-methods design, involving a cross-sectional survey and focus group discussions (FGDs) was used. All midwives (n = 201) working in the maternity sections of the selected hospitals were sampled. Data were collected from February to May 2019 using a self-administered, validated PCMC questionnaire. A sub-set of midwives (n = 56), purposively selected using maximum variation sampling, participated in the FGDs (n = 7). Quantitative data were entered, cleaned, and analysed with SPSS version 20 using descriptive and bivariate statistics and multivariate regression. Statistical significance was set at alpha 0.05 level. Qualitative data were analysed thematically.

**Results:**

The mean age of midwives was 41.8 years ±9.6 years. About 53% of midwives have worked for ≥10 years, while 60% are junior midwives. Overall, the prevalence of low, medium, and high PCMC among midwives were 26%, 49% and 25%. The mean PCMC score was 54.06 (10.99). High perception of PCMC subscales ranged from 6.5% (dignity and respect) to 19% (supportive care). Midwives’ perceived PCMC was not significantly related to any socio-demographic characteristics. Respectful care, empathetic caregiving, prompt initiation of care, paying attention to women, psychosocial support, trust, and altruism enhanced PCMC. In contrast, verbal and physical abuses were common but normalised. Midwives’ weakest components of autonomy and communication were low involvement of women in decision about their care and choice of birthing position. Supportive care was constrained by restrictive policy on birth companion, poor working conditions, and cost of childbirth care.

**Conclusion:**

PCMC is inadequate in public hospitals as seen from midwives’ perspectives. Demographic characteristics of midwives do not seem to play a significant role in midwives’ delivery of PCMC. The study identified areas where midwives must build competencies to deliver PCMC.

## Introduction

Person-centred maternity care (PCMC) is acknowledged as an approach that is critical for achieving improved quality of care during labour and childbirth [1]. PCMC, described as care that is respectful of and responsive to individual women and their families’ preferences, needs, and value, comprises three essential components [2]. The first component is respect and dignity, implying that childbearing women should receive respectful and dignified care. The second, communication and autonomy, entails effective communication with and involvement of childbearing women and their families in care decisions during labour and childbirth. The third is supportive care indicating that maternity care providers should be well-resourced to provide childbearing women emotional support, routine care and manage complications [2, 3]. Maternity care that does not meet these standards limit quality of care, violate childbearing women’s human right, deter them from future facility-based childbirth, and increases the likelihood of maternal death [1, 3–7]. Midwives are important in providing PCMC because they combine technical competencies and proficiency with inter-personal skills and knowledge of organisational structure and its facilities to ensure positive childbirth experiences [8–11].

Global evidence indicates that although implementation of PCMC promotes women’s care-seeking behaviours and positively affects outcomes of received care, women in low-resource settings do not receive adequate PCMC [4, 12]. Several studies underscore a need for midwives to provide childbearing women friendly, safe, dignified, and responsive maternity care with involvement of women in decisions about their care [13–18]. Two key gaps were identified in scholarship; first, policymakers have not been able to provide the conditions that facilitate PCMC; secondly, health care providers have not made PCMC their practice norm [19, 20]. For instance, midwives lack the competencies needed to deliver PCMC [21]. Furthermore, PCMC competencies especially those involving health systems constraints require validation among midwives in different settings [21].

A growing body of studies related to PCMC in African countries highlight context-specific enablers of and gaps in respectful and dignified care, communication and autonomy, and supportive maternity care. Midwives commonly reported verbal abuse, physical abuse, lack of visual privacy, poor record confidentiality, neglect, and non-dignified care [5, 8, 22–33]. Disrespectful care of childbearing women results from lack of co-operation from women [8, 22, 23, 26, 31], lack of resources [5, 22, 23, 28, 29, 34], midwives’ normalisation of abuse [31], negative view of women [27, 31], exertion of power and control over women [8, 24, 25, 30, 31], fear of being blamed for poor childbirth outcomes and medical necessity [5, 8, 22, 23, 26, 31], high workload and tiredness [5, 29, 34], and use of moral judgement [24, 35].

Although some midwives encourage close relationship between midwives and women [28, 36], women involvement in care decisions [32], and women’s choice of childbirth position [36, 37], poor communication and autonomy practices are common in prior African studies. Midwives acknowledged rarely introducing themselves to women [22]; limiting women involvement in decision-making [5, 8, 22, 25, 29, 30, 33, 37]; and discouraging women’s choice of birthing position [5, 22, 35, 38]. Midwives identified language barriers [5, 33, 37]; lack of staff, workload and time pressure [22, 25, 29, 30, 38]; unwillingness of women to communicate their need for help [22, 30]; and women’s non-adherence to medical guidance [22] as drivers of poor communication and autonomy practices.

On supportive maternity care, midwives emphasised lack of labour and childbirth support [22, 39]; inadequate staffing [22, 24, 28–30, 34, 36, 37, 40]; lack of equipment and materials [28–30, 34]; poor motivation and lack of skills [29, 34]; poor access to water [28]; crowded wards [22]; lack of bathrooms [29]; and negative hierarchical relationships with their superiors and doctors [25, 28] as constraints to supportive maternity care in sub-Saharan Africa. Midwives restrict companions during labour and childbirth due to cultural unacceptability, staff or infrastructural constraints [25, 37], negative attitude towards women’s relatives [41] or women’s objection to birth companions [22].

In Nigeria, universal access to comprehensive, quality, maternal health services has been recognised as a health development priority [42]. Despite improvements in facility-based childbirth in Nigeria, maternal mortality rate remain high at 576/100,000 live births [43]. Also, women are dissatisfied with the quality of maternity care [44]. Disrespectful and abusive treatment of women during facility-based childbirth are prevalent [40, 45–48]. In few Nigerian studies involving healthcare providers, verbal abuse, physical abuse, lack of visual privacy, denial of companionship, and disallowing birth position of choice were reported by midwives [40, 45, 47]. High workload, deficient training, concern for positive birth outcome, and lack of incentives were identified provider-level drivers of disrespectful maternity care, while poor work environment such as weak infrastructure and stressful hospital protocols constitute institutional drivers [40, 45, 47, 49].

There is need for more studies on providers’ experiences of PCMC in Nigeria. Existing studies involve a mix of health workers in which midwives are underrepresented. This study differs methodologically from existing Nigerian studies in its use of a validated PCMC scale [2, 18], to explore midwives’ perception of the conditions that facilitate PCMC, and how midwives make PCMC their practice norm. This study, therefore, examined the perspectives of midwives on PCMC in Enugu State, South-east Nigeria. Such evidence will be useful to health decision-makers, managers, and providers to develop interventions to improve responsive and respectful care during childbirth in Nigeria.

## Materials and methods

### Study setting

This study was conducted in seven public hospitals in Enugu metropolis, Enugu State, South-east Nigeria. Enugu metropolis is the capital territory of Enugu State and was selected because of its central location, easy accessibility and number of midwives currently employed. The metropolis comprises three local government areas (LGAs) namely Enugu East, Enugu North and Enugu South LGAs. Enugu East and Enugu South LGAs have each urban and rural populations, while Enugu North is entirely urban. The 2020 population of the metropolis is about 1,093,094 people based on 3% growth rate of 2006 estimate, out of which women of childbearing age constitute 47.2% [43]. In Enugu State, skilled birth attendance is 93%, while about 43.5% of women gave birth in public health facilities [43]. Two university teaching hospitals, two general hospitals and three cottage hospitals serve the metropolis. A total of 201 midwives were working in the maternity sections of the hospitals: 71 in University of Nigeria Teaching Hospital (UNTH), 67 in Enugu State University Teaching Hospital (ESUTH), and 63 in state-owned general and cottage hospitals (SGHs).

### Research design

A sequential explanatory mixed-methods design was used, which involved quantitative (survey-based questionnaire) and qualitative (focus groups) data collection. This approach enabled triangulation of findings.

### Sampling

#### Quantitative

The respondents were all midwives (n = 201), who work in maternity sections of public hospitals in Enugu metropolis at the time of the study. All the midwives were eligible and included in the study (total sampling). Inclusion criteria was working in the maternity section of the hospitals for at least one year preceding the survey and willingness to participate in the study.

#### Qualitative

We purposively selected 56 focus group participants from among the survey respondents reflecting different types of facilities, all LGAs and demographic characteristics of midwives. The midwives must have worked in maternity section of the public hospital for at least one year and willing to participate in the study.

### Data collection tool and data collection

#### Quantitative

Data were collected from the midwives from February to May 2019 using pre-tested, self-administered questionnaire. The questionnaire consists of socio-demographic characteristics and PCMC scale made up of 30 items measuring dignity and respect (6 items), communication and autonomy (9 items), and supportive care (15 items). Each item was measured on a 4-point response scale—0: “no, never,” 1: “yes, a few times,” 2: “yes, most of the time,” and 3: “yes, all the time.” For each midwife, responses from the PCMC scale were summed up into one composite PCMC score. The possible score on the PCMC scale ranges from 0 to 90, with a lower score implying poorer PCMC. The range of possible scores on the sub-PCMC scales are: 0–18, 0–27 and 0–45 for respect and dignity, communication and autonomy, and supportive care correspondingly. The PCMC scale has been validated in similar low-resource context [2, 18]. Among our sample of midwives, Kaiser-Meyer-Olkin measure of sampling adequacy was 0.864 (X^2^ = 2088.38, ρ = 0.000). All 30 items yielded adequate communalities (≥ 0.4) on exploratory factor analysis [50]. The cumulative variance was 60.9%. Oblimin rotation with Kaiser Normalization showed that all 30 items loaded ≥ 0.4 and were retained since a rotated factor loading of 0.32 is considered significant [50]. The reliability coefficient of the PCMC scale was 0.845. The pre-test of the PCMC scale on 10 midwives, working in similar setting, indicated that the questionnaire was easy to understand and took about 30 minutes to complete. Five trained research assistants facilitated administration and collection of questionnaires. We collected 177 (88.1%) questionnaires, of which 168 were appropriate for analysis, resulting in 83.6% net response rate. During the data collection, 11.9% of midwives were unavailable due to leave, training, or shift work.

#### Qualitative

We conducted seven focus group discussions (FGDs) with 56 midwives to gain a detailed understanding of experiences of PCMC and its associated factors. Each focus group comprised 6 to 10 midwives. The FGDs were held at a venue within the hospital and at a time chosen in consultation with the midwives. A topic guide, developed based on the PCMC framework, was used to facilitate the FGDs (Additional file 1). Discussions, which were held in English language and audio recorded with midwives’ consent, lasted 60–90 minutes. Data were collected from February to May 2019 until thematic saturation was reached [51]. We transcribed the audiotapes verbatim, de-identified transcripts and stored them in password protected computer.

### Data analysis

#### Quantitative

Data were analyzed using SPSS (version 20, IBM, New York, USA). Characteristics of respondents were presented using frequencies and percentages. We recoded “no, never and a few times” together and “yes, most of the time and all the time” together to transform the four-point frequency responses to binary responses. The binary responses were analyzed using table, frequencies, and percentages. We categorized full PCMC and each sub-scale into “low, medium and high”. Low was defined as scores in the approximate lower 25th percentile and scores in the top 75th percentile defined as high [52]. Full PCMC and sub-scale scores in each category were also presented using table, frequencies, and percentages. Next, we tested the association of PCMC and sub-scale categories with socio-demographic characteristics of midwives using chi-square. Generalized Linear Models for ordinal scale was used to test relationship between PCMC and the parameters that were significant on bivariate analysis. Statistical significance was set at p < 0.05 level.

#### Qualitative

Characteristics of focus group participants were presented using descriptive statistics. Transcripts were imported into NVivo 11 software and analysed using a thematic framework approach, which involved coding, mapping and organising the data under themes and interpretation [53]. Two persons generated a codebook, read the transcripts, coded the data independently and resolved inter-coder differences by consensus. The themes were largely deduced from dimensions of PCMC, but some sub-themes were generated inductively. Emergent sub-themes reflected factors affecting PCMC. We used excerpts and illustrative quotes to ground our findings in the data.

## Ethical approval and consent

Ethical clearance was obtained from the Health Research Ethics Committee of the University of Nigeria Teaching Hospital, Enugu, Nigeria (NHREC/05/01/2008B-FWA0000258-1RB00002323). Written, informed consent was obtained from each participant. Administrative permission was obtained from heads of hospitals prior to data collection. Data were stored in a passworded computer with access restricted to the research team.

## Results

### Quantitative findings

#### Basic characteristics of study survey respondents

The mean age of midwives was 41.8 years ±9.6 years. Table 1 shows that all midwives were female and Christians. Most midwives were married and Igbo. About 40% were senior midwives while 53% have worked ≥ 10 years. Almost 66% of respondents had bachelor’s degree.

**Table 1:** Basic characteristics of study survey respondents.

Parameters		Frequency (n)	Percent (%)
Gender	Female	168	100.0
Age	20–29	14	8.3
	30–39	64	38.1
	40–39	43	25.6
	50–59	47	28.0
Marital status	Single	26	15.5
	Married	131	78.0
	Others	11	6.5
Ethnic group	Igbo	167	99.4
	Others	1	0.6
Years of service	0–9	79	47.0
	10–19	60	35.7
	≥20	29	17.3
Highest education	Registered midwives	58	34.5
	Bachelors	110	65.5
Cadre	Junior midwives	101	60.1
	Senior midwives	67	39.9
Hospital type	Federal teaching hospital	46	27.4
	State teaching hospital	67	39.9
	State general hospital	55	32.7

#### Distribution of dignity and respect items

Most midwives reported that they treated women with respect, friendly, never abused women verbally or physically, ensured visual privacy and confidentiality of women’s health record (Table 2).

**Table 2:** Distribution of dignity and respect items among midwives.

Item	Response	Frequency (n)	Percent (%)
Treated with respect	Few times or never	23	13.7
	Most or all the time	145	86.3
Friendly	Few times or never	25	14.9
	Most or all the time	143	85.1
Verbal abuse	Never	152	90.5
	At least once	16	9.5
Physical abuse	Never	157	93.5
	At least once	11	6.5
Visual privacy	Few times or never	28	16.7
	Most or all the time	140	83.3
Record confidentiality	Few times or never	22	13.1
	Most or all the time	146	86.9

#### Distribution of communication and autonomy items

As shown in Table 3, the proportion of midwives who reported good communication and autonomy practices ranged from 53.0% to 86.3%.

**Table 3:** Distribution of communication and autonomy items among midwives.

Item	Response	Frequency (n)	Percent (%)
Introduce self	Few times or never	79	47.0
	Most or all the time	89	53.0
Called by name	Few times or never	39	23.2
	Most or all the time	129	76.8
Involvement in care	Few times or never	47	28.0
	Most or all the time	121	72.0
Consent to procedure	Few times or never	30	17.9
	Most or all the time	138	82.1
Delivery position choice	Few times or never	57	33.9
	Most or all the time	111	66.1
Language	Few times or never	23	13.7
	Most or all the time	145	86.3
Explain exam/procedure	Few times or never	33	19.6
	Most or all the time	135	80.4
Explain medicine	Few times or never	35	20.8
	Most or all the time	133	79.2
Able to ask question	Few times or never	31	18.5
	Most or all the time	137	81.5

#### Distribution of supportive care items

Table 4 shows a wide variation in the proportion of midwives who reported good supportive care practices (range,19.0% to 86.3%).

**Table 4:** Distribution of supportive care items among midwives.

Item	Response	Frequency (n)	Percent (%)
Time to care	Few times or never	156	92.9
	Most or all the time	12	7.1
Labour support	Few times or never	89	53.0
	Most or all the time	79	47.0
Childbirth support	Few times or never	108	64.3
	Most or all the time	60	35.7
Talk about feeling	Few times or never	48	28.6
	Most or all the time	120	71.4
Support anxiety	Few times or never	29	17.3
	Most or all the time	139	82.7
Attention when need help	Few times or never	26	15.5
	Most or all the time	142	84.5
Took best care	Few times or never	26	15.5
	Most or all the time	142	84.5
Control pain	Few times or never	44	26.2
	Most or all the time	124	73.8
Trust	Few times or never	31	18.5
	Most or all the time	137	81.5
Enough staff	Few times or never	136	81.0
	Most or all the time	32	19.0
Crowded	Few times or never	78	46.4
	Most or all the time	90	53.6
Cleanliness	Few times or never	70	41.7
	Most or all the time	98	58.3
Clean water	Few times or never	114	67.9
	Most or all the time	54	32.1
Electricity	Few times or never	75	44.6
	Most or all the time	93	55.4
Safe	Few times or never	39	23.2
	Most or all the time	129	76.8

#### Distribution of total person-centred maternity care and subscale

Table 5 shows that few midwives had high perception of PCMC and its subscales. The mean perception of person-centred maternity care is 54.06 (10.99).

**Table 5:** Total person-centred maternity care and sub-scale scores.

Outcome	Frequency (n)	Percent (%)	Mean	Percentiles	
			(SD)	25^th^ 75^th^	
Total PCMC score	N = 168		54.6 (10.99)	47.00	62.75
Low	44	26.2			
Medium	82	48.8			
High	42	25.0			
Dignity and respect sub-scale score	N = 168		10.07 (2.14)	9.00	12.00
Low	63	37.5			
Medium	94	56.0			
High	11	6.5			
Communication and autonomy sub-scale score	N = 168		18.94 (4.99)	16.00	23.00
Low	51	30.4			
Medium	85	50.6			
High	32	19.0			
Supportive care sub-scale score	N = 168		25.05 (5.71)	21.25	29.00
Low	42	25.0			
Medium	95	56.5			
High	31	18.5			

#### Socio-demographic factors associated with PCMC

As shown in Table 6, there was no socio-demographic differences in total person-centred maternity care and across the sub-scales.

**Table 6:** Socio-demographic factors associated with midwives’ perception of person-centred maternity care.

		PCMC		DR		CA		SC	
Parameter		Mean	SD	Mean	SD	Mean	SD	Mean	SD
Age	20–29	50.1	8.3	9.9	1.9	17.7	4.5	22.6	3.7
	30–39	53.9	11.3	10.0	2.2	18.6	5.3	25.2	5.9
	40–49	55.4	10.3	10.4	1.9	19.3	4.3	25.6	5.4
	50–59	54.3	11.8	9.8	2.4	19.4	5.3	25.0	6.2
	ρ-value^1^	0.488		0.603		0.607		0.372	
Marital status	Single	51.0	10.1	9.6	2.3	18.0	4.0	23.4	5.5
	Married	55.0	10.5	10.2	2.0	19.2	5.0	25.5	5.6
	Others	49.7	16.1	9.0	3.3	17.5	7.0	23.2	6.7
	ρ-value^1^	0.096		0.091		0.323		0.120	
Ethnicity	Igbo	54.1	11.0	10.1	2.1	19.0	5.0	25.1	5.7
	Other	40.0	0.0	8.0	0.0	15.0	0.0	17.0	0.0
	ρ-value^2^	0.200		0.335		0.429		0.158	
Years of service	0–9	54.7	9.3	10.3	2.0	19.1	4.5	25.3	4.9
	10–19	54.6	12.3	10.1	2.1	19.1	5.3	25.4	6.8
	20–35	51.3	12.3	9.3	2.5	18.2	5.7	23.8	5.4
	ρ-value^1^	0.326		0.099		0.661		0.423	
Educational attainment	Registered midwife	55.5	9.5	10.3	1.8	19.3	4.8	25.8	5.0
	Bachelors	53.3	11.7	9.9	2.3	18.7	5.1	24.7	6.0
	ρ-value^2^	0.229		0.251		0.447		0.244	
Cadre	Junior midwife	54.5	10.1	10.2	2.0	19.1	4.9	25.1	5.2
	Senior midwife	53.4	12.3	9.8	2.4	18.7	5.2	24.9	6.4
	ρ-value^2^	0.539		0.259		0.571		0.792	
Hospital type	Federal teaching hospital	54.3	9.0	10.3	2.1	19.8	4.1	24.3	5.0
	State teaching hospital	53.0	10.9	10.0	2.0	18.2	5.1	24.9	5.4
	State general hospital	55.1	12.5	10.0	2.4	19.2	5.4	25.9	6.5
	ρ-value^1^	0.583		0.677		0.222		0.356	

^1^According to ANOVA

^2^According to t-test PCMC = person-centred maternity care.

DR = dignity and respect; CA = communication and autonomy; SC = supportive care.

Gender and religion were not included because the sample was entirely female and Christian.

#### Predictors of person-centred maternity care among midwives

Table 7 shows that midwives’ perception of PCMC was not significantly related to socio-demographic factors.

**Table 7:** Predictors of midwives’ perception of person-centred maternity care.

Parameter		B	95% Wald Confidence Interval		
			Lower	Upper	ρ value
	(Intercept)	38.8	16.7	60.8	0.001
Age	20–29	-6.9	-15.7	1.9	0.126
	30–39	-4.8	-10.9	1.4	0.127
	40–49	-0.1	-4.9	4.6	0.959
	50–59	0^a^			
Marital status	Single	-0.2	-8.4	8.1	0.969
	Married	3.3	-3.6	10.2	0.344
	Other	0^a^			
Ethnicity	Igbo	10.4	-12.4	33.3	0.370
	Other	0^a^			
Years of service	0–9	4.6	-1.1	10.2	0.113
	10–19	3.1	-1.9	8.1	0.229
	≥ 20	0^a^			
Education attainment	Registered Midwife	2.2	-1.7	6.1	0.268
	Bachelors	0^a^			
Cadre	Junior midwife	3.5	-2.4	9.4	0.242
	Senior midwife	0^a^			
Hospital type	Federal teaching hospital	-0.9	-5.5	3.7	0.699
	State teaching hospital	-2.8	-7.1	1.5	0.196
	State general hospital	0^a^			
	(Scale)	109.0^b^	88.0	135.0	

^a^Set to zero because this parameter is redundant.

^b^Maximum likelihood estimate.

Gender and religion were not included because the sample was entirely female and Christian.

### Qualitative findings

#### Basic characteristics of focus group participants

Table 8 shows that all participants were female, Igbo, and Christians. Most midwives were age ≤ 40 years, married, employed in state government hospitals and tenured for ≤10 years. Almost 54% of were junior midwives.

**Table 8:** Basic characteristics of focus group participants.

Parameters		Frequency (n)	Percent (%)
Gender	Female	56	100.0
Age	20–29	6	10.7
	30–39	16	28.6
	40–49	15	26.8
	50–59	19	33.9
Marital status	Single	11	19.6
	Married	43	76.8
	Others	2	3.6
Years of service	0–9	24	42.9
	10–19	18	32.1
	≥20	14	25.0
Educational attainment	Registered midwives	22	39.3
	Bachelors	34	60.7
Cadre	Junior midwives	30	53.6
	Senior midwives	26	46.4
Hospital type	Federal teaching hospital	14	25.0
	State teaching hospital	18	32.1
	State general hospital	24	42.9

#### Dignity and respect

Most midwives stated that they treated all women alike irrespective of their personal attributes. Few midwives, however, noted that government officials and wealthy people get preferential treatment from providers as a mark of respect, or in anticipation of pecuniary benefits. *“If you look wealthy*, *everybody will flood your bed even when they do not have any role in your care*, *but if you look poor*, *nobody will come to you*. *Even when you come to the nursing station to ask for something*, *nurses may shout at you*” (M1 FGD4, TH). One midwife remarked *“If her excellency [wife of the governor] comes here to deliver*, *… junior nurses will stay clear and let older nurses attend to her”* (M6, FGD7, GH). Equally, persons living with HIV expect to be discriminated: *“They watch us with critical eye to see whether there is an atom of discrimination*, *but here we treat all women alike”* (M7, FGD3, TH)

Most midwives observed that they treated women in a friendly manner by *“creating a therapeutic environment”* (M6 FGD4, TH) through care that is *“individualised*, *appropriate*, *acceptable and safe”* (M7 FGD4, TH). Midwives attributed unfriendliness to quacks, who are not trained: *“every woman sees anybody on white as a nurse and green*, *as a midwife*. *So*, *any ill treatment they have received*, *they think it is from the midwife”* (M6, FGD1, TH).

All midwives acknowledged that verbal abuse is common. However, many midwives viewed it as *“verbal encouragement because though what you say to the women may sound harsh*, *it will spur them to take action that leads to a positive outcome”* (M6, FGD3, TH). Midwives identified five factors that precipitate verbal abuse. First, some women come to the hospital without any birthing materials. Secondly, when women refuse to disclose their HIV status, midwives normally frown at that. Thirdly, midwives are unhappy with women who default after antenatal care registration and re-present during labour. Fourthly, some women lack good personal hygiene: *“some women come to the hospital without taking their bath*. *It’s annoying”* (M3, FGD5, GH). Finally, non-adherence to midwife’s instructions by women or their relatives. Midwives explained that they scold women to elicit their co-operation for a positive birth outcome: *“if the woman gets up or closes her legs when they head of the baby is already out*, *you must use a harsh tone to bring the woman down”* (M9, FGD2, TH).

Most midwives mentioned that they would strongly pat uncooperative women on the laps to open their legs when *“the baby is about come out and the woman is persistently closing her legs”* (M5, FGD1, TH) because *“when things go wrong*, *the person that is usually blamed is the birth attendant”* (M8, FGD1, TH). Moreover, *“for a midwife*, *to have a stillbirth is a dilemma that one lives with all through one’s life”* (M3, FGD3, TH). In contrast, few midwives insisted that *“beating or spanking a woman in labour is an abuse as there are so many ways one can assist a woman to deliver her baby”* (M2 FGD4, TH). It is also an abuse *“when a midwife uses needle to prick an unmarried adolescent”* (M6 FGD2, TH) during childbirth.

Almost all midwives reported that visual privacy was achieved using screens and curtains; and patients’ records are kept strictly confidential. Even for HIV positive woman, when a woman says, *“I do not want my husband to know about this”* (M4 FGD4, TH), the midwife will not disclose her HIV status to her husband.

#### Communication and autonomy

While many midwives said that they introduced themselves appropriately to women, some midwives indicated that “as for introducing ourselves, we do not always do that” (M3 FGD4, TH). It was explained that *“we are conversant with them*. *We live with them*. *We are familiar with them*. *We know ourselves*. *They will even call you on the phone before coming to the hospital”* (M6, FGD7, GH). Also, *“some midwives do not like women to know their names”* (M8, FGD1, TH) or assume that *“once the woman sees me on white*, *she knows am the nurse on duty”* (M1, FGD5, GH) or introduction are limited by workload.

Many midwives also stated that they called women by their names. Even though *“it is a taboo for you not to call a woman by her name”* (M8, FGD1, TH), “some midwives addressed women as “Madam” (M2, FGD1, TH). Also, high workload constrained midwives from calling women by their name: “*we do not usually call them by their names due to high workload”* (M6, FGD5, GH).

Midwives were divided on involvement of women and their relatives in decisions around their care. Although some midwives mentioned that *“we give individualized care and respect their autonomy as long as mother and baby are safe”* (M4, FGD3, TH), others noted that *“in this part of the world*, *it is mostly healthcare providers that make delivery decisions*. *Even with some enlightened women*, *after educating them*, *they will still come back to you with ‘nurse what do you want me to do’*?*”* (M3 FGD4, TH). Women involvement was limited when midwives consider women’s requests as unnecessary. For instance, when a woman says, *“I do not give birth without hot drip”* (M3, FGD 7, GH), but there is no clinical indication for augmenting the labour. Also, some women greeted the news of assisted delivery with such notions as *“God forbid”* (M3, FGD 7, GH), *“not my portion”* (M2, FGD7, GH) and *“no*, *my pastor said I must give birth like a Hebrew woman”* (M1, FGD3, TH). In such circumstances, midwives do not wait *“for the woman and her spirituality”* (M7, FGD1, TH) to take appropriate actions.

Most midwives noted that women were not always involved in decisions about their birthing position: *“No*, *you are the one who will tell them the position*. *I want you to stay like this*, *so that proper decent will be attained during labour”* (M1, FGD7, GH). Even when *“there are people who like to stand up at labour because they feel less pain doing so*. *We cannot leave them to give birth standing up*. *So*, *we force them to bed”* (M10, FGD1, TH). Conversely, some women insisted on their birthing position: *“despite all our pleas*, *a Hausa woman refused to lie down*. *She squatted until she had her baby”* (M5, FGD6, GH). Similarly, a nurse preferred to give birth while standing up: *“She stood on the bed and she gave birth standing up”* (M8, FGD2, TH).

Most midwives elicited consent from women, based on questioning by and explanations of care processes to women in the language the women understood. Midwives explained that when a woman signs consent on admission, she *“agrees to all procedures that will be done to her during labour and childbirth”* (M7, FGD3, TH). Yet, *“whatever procedure we are carrying out*, *we explain it to the woman and get her go-ahead”* (M3, FGD6, GH). However, some midwives remarked that *“we do not usually explain the procedure to the woman and most times we do not even wait for them to respond*. *We just say*: *madam*, *I want to do this*, *and we just go ahead”* (M2, FGD1, TH).

#### Supportive care

Most midwives stated that they pay attention to women, empathise with them, provide psychological support and control women’s pain most time. Constant reassurance, counselling, encouraging women to take deep breathing exercises, walking and massaging were common approaches midwives employed to control pain during labour. Additionally, asking women *“to call their pastor*, *reverend or imam to pray or talk to them helped to allay their anxiety”* (M3, FGD5, GH). However, some midwives noted *“lack staff to attend to many women needing attention at the same time”* (M6, FGD3, TH) as a constraint.

Most midwives revealed that allowing labour and childbirth support was not the norm because *“there are other mothers in labour and sometimes when you advise a woman on what to do*, *the relation will tell the woman a different thing”* (M9, FGD2, TH) or *“they start teaching you your job”* (M4, FGD5, GH). Nonetheless, relatives of women were allowed into labour or birthing room under exceptional circumstances. First, lack of manpower: *“when you are alone conducting childbirth and the support person is an experience woman*, *you can allow her to be around and assist you”* (M2, FGD7, GH). Secondly, to avoid blame for a poor childbirth outcome: *“when the woman is not co-operating during childbirth*, *we bring in the relative*, *even the husband*, *to talk to her and to bear witness in case the childbirth ends negatively”* (M3, FGD2, TH). Thirdly, to facilitate referral of women who experience complications. Fourthly, to provide needed items that are unavailable in the health facility. However, *“many times women do not want their husband to be around”* (M6, FGD3, TH).

Midwives did not consider the maternity section clean, safe and health always. In some hospitals, *“the wards are very dirty*. *We had a case where the relative of a woman in labour was bitten by scorpion one night”* (M6, FGD7, GH). Epileptic power supply, poor water supply and inadequate toilet facilities were common problems in maternity sections: *“one major problem this hospital is facing is lack of toilet facility*. *If you go to the labour ward*, *we have only one toilet”* (M9, FGD1, TH). Where toilets exist, midwives blamed women for not keeping them clean: *“some dispose their pads in the toilets*, *while others simply mess up the place”* (M4, FGD6, GH). Yet, hospitals lacked staff responsible for cleaning toilets: *“we have only one staff*, *she cannot run three shifts for seven days in a week”* (M4, FGD5, GH).

Midwives believed that women trusted them and that they took the best care of women. However, labour wards are crowded, and hospitals lack enough staff. In tertiary hospitals doctors and midwives work as a team, but in general hospitals midwives seem to bear higher burden of the high workload as doctors were not always available when needed: *“Am not going to talk about doctors who are supposed to be around*. *Here*, *it is only midwives all the time”* (M5, FGD7, GH). Another midwife corroborated, *“the midwife belongs to every department*. *She finds card; she admits the patient; she is the doctor; she is the pharmacist; and she is the midwife”* (M3, FGD5, GH). Furthermore, in state-owned hospitals, exit of junior midwives due to inadequate compensation exacerbate the workload of remaining midwives.

Most midwives observed that the cost of maternal care is high: *“The cost of everything in this hospital is very high*, *considering that many people that patronize us are poor people”* (M8, FGD2, TH). It was explained that “*most things have been commercialized and patients must provide the necessary things needed for their care”* (M1, FGD1, TH). Women also pay for drugs that were covered by free care policy. Many midwives revealed that women who are unable to pay their bills are detained in tertiary hospitals for several weeks until they pay. Detained women are usually un-booked patients who are brought as emergencies and needing surgery. Often, *“these women are operated on loans–drugs on loan*, *blood on loan*, *everything on loan–and there is no money to pay for them”* (M5, FGD3, TH). Midwives involve security men in monitoring women *“because if you are on duty and a woman absconds*, *the hospital management will query you”* (M5, FGD2, TH). Few midwives observed that in some cases, midwives engage with social workers to assist detained women pay the bills. For women who attend antenatal care, midwives link high-risk women to national health insurance scheme: “*if we check the history and there is a possibility of delivering via Caesarean section*, *we do advice the mother to enrol into national health insurance”* (M4, FGD2, TH). Additionally, midwives use drugs from emergency drug cupboards in labour and post-natal wards and get refund from women. Midwives reported that women often misconstrued these cash payments as under-the-table payments. Furthermore, midwives occasionally mobilise private funds to help poor women.

## Discussion

This study examined midwives’ perceptions of PCMC and the conditions that facilitate PCMC in public hospitals. Three key areas emerge from the study that needs to be explored further. The first is the low proportion of midwives with high perception of PCMC. The second is the fact that midwives’ perception of PCMC was not significantly related to socio-demographic factors. The third factor is the better understanding of practices and contextual drivers of respect and dignity, communication and autonomy, and supportive care during childbirth. All these factors indicate a need to improve midwives’ practice of PCMC given that women’s perceptions of PCMC strongly influence their choice of birthing facilities [4–7].

Our findings of low mean PCMC score and low proportion of midwives with high perception of PCMC implies that most midwives do not provide women adequate person-centred care during facility-based childbirth. This finding aligns with high prevalence of mistreatment and disrespectful care among midwives in prior studies in South Africa, Nigeria, and Tanzania [33, 47, 54]. In our study, respect and dignity domain had the least proportion of midwives with high perception, followed by supportive care, and communication and autonomy. Mostly, when compared to childbearing women [55], a higher proportion of midwives reported positively on PCMC items than childbearing women. A similar pattern of discordance of PCMC measures for women and providers was found in Kenya [22]. Consequently, our finding not only confirms the poor PCMC reported by women in our study setting [55], it also underscores a need for midwives to institutionalise PCMC practices [19, 20] by building the competencies needed to deliver PCMC [21, 31].

The finding that midwives’ perception of PCMC was not significantly related to any socio-demographic characteristics means that current PCMC practices involve all midwives irrespective of their individual or job-related characteristics. We expected from previous Nigerian studies that poor PCMC practices would be associated with older, single, and junior midwives [47, 49]. Differing methodological approaches might explain the variation in the studies. Our study differed from the prior studies in two ways. First, participants in the earlier studies were mostly doctors and other stakeholders; we studied only midwives. Secondly, unlike our study that involved both tertiary, general and cottage hospitals, the previous studies were limited to a single tertiary hospital and /or private(mission) hospital.

Despite a discordance in self-reported verbal and physical abuse between the quantitative and qualitative components of our study, our qualitative findings that verbal and physical abuse were common among midwives is similar to results of preceding studies [5, 8, 15, 22–28, 40, 45, 47, 54]. The discordance in this study might, as reported in a previous Nigerian study, be due to self-reporting bias in accounts of witnessing and enacting abuse during childbirth [47]. It may well be due to normalization of verbal and physical abuse based on cultural expectation, norm, and value of midwives’ responsibilities [31]. While Kenyan midwives were more likely to report verbal abuse than midwives in our study, our low prevalence of physical abuse compares to evidence from Kenya [22]. As in prior studies, midwives rationalised verbal and physical abuse as encouragement, necessary to save the lives of mother and newborn, and avoid being blamed for poor childbirth outcomes [5, 8, 22, 23, 26, 31, 40, 45]. To promote respectful care, midwives require interpersonal competencies to handle women’s lack of birth preparedness, late presentation, poor hygiene, refusal to disclose HIV status, and non-adherence to midwife’s instructions, which commonly drive verbal and physical abuse. Hence, a re-orientation of existing midwives and re-design of midwifery curriculum is warranted.

Midwives’ weakest components of autonomy and communication were low involvement of women in decision about their care and choice of birthing position. These are consistent with evidence that midwives dominated decision making process in care and birth position [5, 8, 22, 25, 29, 30, 33, 35, 37, 38, 47], but contrasts with studies wherein midwives encouraged women involvement in caring decisions and choice of childbirth position [32, 36, 37]. As similarly found in previous studies, there were power imbalances between midwives and women, such that women always relied on midwives for caring decisions [30, 49]. Also, when midwives focus on clinical functions and death avoidance, they limit provision of information to women and promote specific behaviours including birthing positions [5, 22, 25, 29, 31, 33, 37]. For instance, midwives disregarded inappropriate and unrealistic choices by women which have no clinical basis, as found in previous studies [25, 37]. Moreover, like findings of other studies, workload and time pressure limited communication between midwives and women [22, 25, 29, 38]. In order to change, midwives must promote an environment of shared power and responsibility between women and midwives to ensure positive childbirth experiences and satisfaction with maternity care [3, 8–10].

Furthermore, the study highlighted the role of birth companionship, work context, and altruism among midwives in promoting PCMC. The finding that restriction of birth companion was the norm is consistent with results of prior studies [15, 22, 25, 33, 37, 39]. Midwives do not perceive birth companionship to be necessary except where health facilities lack staff or in emergency situations when midwives needed a witness to avoid being blamed for a poor outcome. Comparable to existing evidence, infrastructural deficits and the need to maintain visual privacy [33, 56], negative attitude of midwives towards women’s relatives [41] and unwillingness of women to accept birth companions [22] were contextual drivers of restrictive policy on birth companionship. Similarly, other researchers have reported exceptions to restrictive labour and childbirth support [22, 39].

This study revealed that work conditions and system factors that constrain person-centred maternity care. Public hospitals lack enough midwives and working materials similar to findings of past studies [24, 28, 29, 34, 36, 37, 40], which meant that midwives have to work long hours under difficult conditions. In addition, midwives were poorly motivated due to poor salaries, lack of support from doctors, and negative hierarchical relationships with doctors as shown by several studies [8, 28, 29, 34, 38]. Our findings that maternity wards are always not clean due to overcrowding, poor access to water, lack of toilets, insufficient cleaning staff, and unsanitary practices by women also agree with existing evidence [22, 28, 29, 40]. Addressing these health system and organisational constraints would improve working condition and environment of midwives and contribute to effectiveness of PCMC interventions.

High cost of care emerged, in this study, as a significant threat to midwives’ delivery of PCMC especially in tertiary hospitals, where women are detained for several weeks for inability to pay their bills. This finding confirms the high prevalence of detention of women for failure to pay their childbirth bills in Nigeria [46]. Relatedly, women must buy some items needed for their care outside the health facility despite existing free maternal and child healthcare programme. A prior study found that women, who expect to receive free childbirth services, are dissatisfied when providers request them to purchase medications [15]. The experiences of midwives in this study offer lessons on several coping strategies which can be adopted in low-resource settings to ensure positive childbirth experiences. One option is for midwives to identify and link high-risk women to social protection schemes in the course of antenatal care. A second alternative is for midwives to borrow drugs and supplies from the health facility during childbirth and get refund from women afterwards. Midwives might also mobilise private funds to make altruistic payments for poor women. As cost of care makes it difficult for midwives to practice PCMC, it would be helpful to prioritise maternal health in universal coverage schemes in public hospitals.

This study contributes to scholarship on providers’ experiences of PCMC in resource-limited settings. This is one of the few studies reporting midwives’ perceptions of PCMC from three levels care–tertiary, general and cottage hospitals. Understanding poor PCMC practices among midwives and why disrespectful and unresponsive maternity care happens is an important contribution to evidence-informed maternal health policy. Although the specific findings may not be representative of the entire Nigeria, evidence from this study could be used to develop interventions to improve midwife-led care of women during childbirth in our study setting. The effect of these interventions can be assessed in future studies. Nevertheless, our findings could be potentially limited by social desirability bias as midwives’ perspectives of PCMC might not always reflect actual practice. However, the use of focus group discussion encouraged debate among midwives when it seemed that some midwives described their practices in positive light. Moreover, in the quantitative part of this study, the scope of factors related to midwives’ perception of PCMC excluded some job-related factors such as workload, length of shifts, supervision, interaction with co-workers, and professional status, which should be subject of future studies. Finally, as community health extension workers mostly provide maternity services in primary health centres in Enugu, Nigeria, future research on PCMC should target them.

## Conclusion

PCMC is inadequate in public hospitals, as seen from midwives’ perspective. Overall, few midwives had high perception of PCMC. The proportion of midwives with high PCMC was least in the ‘dignity and respect’ domain. Demographic characteristics of midwives do not seem to play a significant role in the practice of PCMC. Verbal and physical abuses were common but normalised. Midwives’ weakest components of autonomy and communication were low involvement of women in decision about their care and choice of birthing position. The study further provides insights into the role of birth companionship, work context, and altruism among midwives in promoting PCMC. In conclusion, improving delivery of PCMC among midwives, therefore, requires interventions across the three domains of PCMC.

## Supporting information

S1 File Appendix A: FGD guide. (DOCX)
