# Supplementary material for: Midwives’ perspectives on person-centred maternity care in public hospitals in South-east Nigeria: A mixed-method study
Source: PLoS One. 2021 Dec 10;16(12):e0261147. doi: 10.1371/journal.pone.0261147 (PMC8664165; doi:10.1371/journal.pone.0261147)
Supplement: S1 File — (DOCX) [file pone.0261147.s001.docx]

**Appendix A: FGD guide for midwives**

| **S/N** | **Key issues** |
| --- | --- |
|  | What do you understand as patient-centred maternity care? |
|  | Why do you think patient-centred care is important? Probe for   1. Freedom from physical abuse 2. Right to information, informed consent and refusal and respect for choices and preferences 3. Confidentiality and privacy 4. Dignity and respect/ freedom from verbal abuse 5. Equitable, freedom from discrimination based on specific attributes 6. Right to timely healthcare and to highest attainable level of health 7. Freedom from detention in facilities. |
|  | How do midwives talk to mothers during labour and delivery care? Probe for midwives using   1. harsh tones or shouting 2. undignified language 3. threats if mothers don’t cooperate |
|  | Some midwives slap/pinch/hit/beat, stitch episiotomy without anaesthesia, or touch women inappropriately during examination? Yet, some women are free from physical harm and ill treatment? What have you observed? |
|  | How common are harmful practices in maternity units? Probe for   1. excess of vaginal examination, 2. unnecessary separation of mother and newborn after delivery, 3. unhygienic practices- bed sharing, no change of linen, several babies sharing incubator, dirty environment etc. 4. Inappropriate use or overuse of drugs and technology (like episiotomy, etc) |
|  | How common are discrimination based on specific attributes: mothers’ age, marital status, ethnicity, race, HIV status or economic status? Probe for   1. worse treatment of younger or unmarried mothers, 2. detention of the woman in facility due to lack of payment of facility fees. 3. failure to provide services due to personal values. |
|  | How common are abandonment/neglect of care of mother a problem in health facilities? Probe for   1. time to care 2. mother left unattended, 3. mother ignored while asking for pain relief/medication; 4. failure to offer services even when staff are adequate; 5. failure to examine mothers according to guidelines even when the resources are available). |
|  | How do midwives share women’s health information or medical results? Probe for information shared when others could hear? |
|  | How are women involved in decisions about labour and delivery care in health facilities? |
|  | Procedures conducted without consent (permission to undertake procedures diagnosis, progress, results and options provided to patients e.g. medicines, examinations. |
|  | Midwives introduce themselves when they first came to see you; explanations on scope of services or procedures; Information given in an open and friendly manner; clients encouraged to ask questions) |
|  | Cultural birth preferences & medically unjustified and culturally insensitive restrictions on the mother (such as denying drink and food during labour, denying liberty of movement during labour, denying choice of position for delivery). |
|  | How do family and friends support women during labour and delivery in health facilities? |
|  | How do midwives support women and during labour and delivery?   - - 1. Talk about feeling     2. Support anxiety     3. Control of pain     4. Attention when needed     5. Took best care |
|  | What do you think about the facility environment?   1. Enough staff 2. Crowded 3. Clean 4. Utility supply – water, electricity? 5. Safe 6. Informal payments from you or family 7. Availability of drugs and supplies |
|  | Some people say that providing a mom and baby make it through labour and delivery alive, that is all that matters. What do you think about this?  Probe for workload, personalities of midwives, disrespect from patients, culture of blaming midwives for poor outcomes, no alternative to abuse. |
|  | What factors contribute to disrespect and abuse during labour and delivery care?  What strategies would be helpful to improve patient-centred maternity care |
